# Mr. Franks, on Variolous Contagion

**Published:** 1801-02

**Authors:** John Franks

**Affiliations:** Smith Street, Westminster


### *Mr. Franks, on Variolous Contagion.*

[Continued from pp. 81—86 of our laſt.]

To theſe teſtimonies, the number of which might have been greatly augmented, Dr. Watkinſon obſerves, I ſhall beg leave to ſubjoin my own.— " I have paid particular attention to the point in queſtion, ſince the eſtabliſhment of the Diſpenſary for general Inoculation; and can with truth affirm, that a ſingle inſtance has not yet occurred in that charity, in which the contagion has been ſpread by an inoculated patient. Where the chance of ſpreading it has been apparently great, I have been very ſtrict in my inquiries. In many caſes the circumſtances have been ſuch, that if the apprehenſions of a celebrated Inoculator were well founded, the diſtemper muſt inevitably have been communicated.

" Some have been inoculated in narrow ſtreets, in the midſt of thoſe who were obnoxious to the Small-pox, and others in little courts, where, according to the common opinion, the danger of communicating the diſeaſe was ſtill greater.

" In the latter caſe, the patient has ſometimes been kept in a little room on the ground floor, the door of which opened directly into the court, and in the day time was ſeldom ſhut. Before this door, and within a few yards of the perſon inoculated, a number of children have continued to play during the whole courſe of the diſorder, and, as has been already affirmed, without receiving the infection.

" Baron Dimſdale indeed aſſerts, that inſtances of a contrary kind have frequently fallen within his obſervation. But, as the Baron does not ſeem to have been aware of the great influence of the epidemic conſtitution of the air—it is poſſible that what he ſhould have attributed to this cauſe, he has ſometimes imputed to ſimple contagion. Be that as it may, a writer in the *Monthly Ledger*, under the ſignature of J. S. who had an opportunity of ſeeing the practice of inoculation in the country where theſe inſtances happened, ſpeaks of the conſequences of it in a language very different from that which is held by the Baron.

" Dr. Tiſſott, in a piece intitled, *L'Inoculation juſtifiée*, very juſtly obſerves, that the Small-pox is indeed a contagious diſeaſe; but, that it does not propagate itſelf ſo much by contagion as by an *infection of the air*, produced by cauſes which are unknown to us.

" The truth of this obſervation is exemplified in a very ſtriking manner, by a fact which is related by Dr. James Sims in his *Obſervations on Epidemic Diſorders.*

" About

"About the autumnal equinox," ſays he, "bilious diſorders declined, giving way to the Small-pox, that with unheard of havock deſolated the cloſe of this year, and the ſucceeding ſpring of 1767. They had appeared above a year before, along the eaſtern coaſt of the kingdom, and proceeded ſlowly weſtward with ſo even a pace, that a curious perſon might with eaſe have computed the rate of their progreſs. In this they were ſcarcely to be interrupted, as appeared by the following inſtance. The children of ſoldiers on their march, had brought them from other places to ſome towns here, during the preceding ſummer; and although they were of a malignant kind, the afflicted all dying, and therefore moſt fit to propagate the infection, yet *not one of the inhabitants received them, until, in their regular progreſs, they had travelled over the intermediate ſpace.*

"It appears from the *Premier Rapport ſur l'Inoculation* of Dr. Petit, that the Hotel Dieu, a large hoſpital in the centre of Paris, is never free from the Small-pox, and that at certain times the ward deſtined to receive thoſe who are ſeized with that diſeaſe is extremely full; that notwithſtanding the multitude thus crowded together, and the enormous quantity of infection produced, and that in a place too which is open to the public, and where there is continually an immenſe concourſe of all ſorts of people, the diſeaſe is not obſerved to be always preſent in the neighbourhood of this hoſpital, nor even to be more common there than in other parts of the city.

"A remarkable inſtance of the inſufficiency of contagion alone to the production of this diſtemper is authenticated by Dr. Sandifort, the preſent Profeſſor of Anatomy and Surgery in the Univerſity of Leyden.

"One of the children in the Orphan-houſe at the Hague was ſeized with the Small-pox; and though the communication between the patient and the reſt of the orphans was not interrupted, none of them caught the diſeaſe.

"Another inſtance of the ſame kind, and not leſs remarkable, I remember to have heard related by the celebrated Profeſſor Van Doeveren of Leyden, in his lectures on the practice of medicine.

In the ſpring of the year 1762, a company of foot, with twelve children labouring under the Small-pox, entered the city of Groningen, which was then entirely free from that diſeaſe. Theſe children were diſperſed in the houſes of the poorer ſort of inhabitants, in the midſt of numbers who had not had the diſtemper, and who, conſtrained by their poverty, could not fly from its approach.—A fairer trial of the power of ſimple contagion could ſcarcely have been deviſed. The event

 was was fuch as convinced the learned Profeffor that this power was inadequate to the effects which had been commonly afcribed to it.—The epidemic threatened was not produced, nor, which is more extraordinary, was the diforder propagated fporadically; for, after the moft fedulous inquiry, not an individual could be found to whom the infection had been communicated.

" To multiply facts of this fort, Dr. Watkinfon obferves, would be eafy, but I truft that thefe will be quite fufficient to eftablifh the pofition for which they were brought. The Doctor therefore quits this ground, and meets the Anti-Inoculifts upon that in which they have hitherto thought themfelves fecure of victory, namely the Bills of Mortality. And in this refpect the Doctor appears to be no lefs happy, for he has fhewn, that although the prevalence of inoculation and the increafed mortality of the Small-pox, may at fome particular periods have coincided, (for according to the laws of chance, this muft fometimes have happened) yet, from the great irregularity of their coincidence, it may be fairly inferred, that *they are not connected together as caufe and effect.* And upon this fact, the Dr. obferves, " the defence of inoculation againft the charge of increafing the mortality of the Small-pox, might perhaps be fafely refted; but I fhall add to it another, derived likewife from the Bills of Mortality, which appears to be conclufive. For the fact alluded to, I am indebted to my ingenious friend Dr. James Sims.

" An objection has been made to inoculation, and lately fupported with confiderable warmth by feveral very refpectable writers, which, if founded in truth, would be fufficient to prove that the practice of this art is detrimental to fociety. It is afferted, that, by inoculation the contagion is fo much propagated, that the victims to the Small-pox have been more numerous fince than they were before that practice obtained; and that the mortality has increafed in proportion to the reception of the art.

" To prove thefe affertions, it has been ufual to extract the deaths by the Small-pox from the Bills of Mortality, for a certain number of years previous to the introduction of inoculation; and to compare the general average of thefe with the average of deaths fince that time; and by dividing the latter into feparate periods of years, to fhew, that the proportion of deaths by the Small-pox has been conftantly increafing fince the practice of inoculation began.

" Thus, one writer, who gives a view of the Bills of Mortality for eighty-four years, fhews, that in forty-two years previous to inoculation, only feventy-two deaths in every thoufand were owing to the Small-pox; whereas, in forty-two years afterwards, afterwards, the deaths by that difeafe amounted to eighty-nine in every thoufand; and that, by dividing the latter of thefe into leffer periods, the average of deaths is as follows:—In the firft twelve years it is feventy-four in a thoufand; in the next ten, eighty-three; in the next, ninety-fix; and in the laft ten, one hundred and nine.

"This conftant increafe is attributed to inoculation; and the argument appears to be properly ftated, as it guards againft any deception which might arife from the variations in the general number of deaths: I have endeavoured to ftate this objection in the ftrongeft manner, and hope that I fhall be able to give a decifive anfwer to it.

"The reafon why the above objection has not hitherto been fatisfactorily anfwered, is this; thofe who have attempted it, have taken the Bills of Mortality as garbled and unfairly ftated by the objectors to inoculation, without giving themfelves the trouble of further examination.

"The circumftance in which the objectors have dealt unfairly by us is, that in taking the medium of deaths for a certain number of years prior to the practice of inoculation, as a fixed ftandard, they have not once hinted that the mortality of the Small-pox had increafed in the fame proportion before, as it has done fince the introduction of that art; and by prudently publifhing only a part of the Bills, they have given us no opportunity of making this difcovery. Had they given the whole of the Bills, is it to be fuppofed that any man in his fenfes would have joined with them in blaming inoculation for an increafe, *which commenced ninety years before inoculation was heard of in this country, and continued progreffive through the whole of that period?*" Dr. Sims has conftructed eight tables; thefe tables will fhew that the mortality from cafual Small-pox, inftead of increafing, is at prefent (*id. eft.* more than twenty years ago) confiderably declining.

Thus have I extracted from Dr. Watkinfon's work, a body of evidence in fupport of the opinion which I fubmitted to the readers of the Medical and Phyfical Journal in a former number, namely, that the fuperiority of the Vaccine over the Variolous Inoculation is fomewhat over rated; by fhewing that the accufation which has been brought againft inoculated Small-pox, of propagating contagion, and increafing the mortality of cafual Small-pox, is void of foundation.

Had it not been for this unfortunate objection, the inftitution for inoculating the poor of London, at their own habitations, would have continued to exert its benign influence, and ere this have refcued thoufands, who may have fell victims to the malignity of Small-pox; but it was reprefented as fraught with with very dangerous confequences to the community, and as tending, by fpreading the contagion, to increafe the very evil it was defigned to leffen.

Until a fufficient number of fimilar inftitutions are eftablifhed in London, to inoculate the whole of the poor with vaccine or variolous matter, as each individual may prefer, let the Minifters in every parifh be enjoined, by proclamation if expedient, to take opportunities occafionally to recommend the inoculations for univerfal adoption, by pointing out to their audiences the incalculable benefits which would arife from fuch a procedure. Let thefe recommendations from the pulpit be feconded by the governors of the poor;—let it not become a parifh job;—let not the poor's rate be increafed on this account;—let not the concern be entrufted to one or two individuals in a parifh; but let every medical perfon refident in the parifh be invited to lend his affiftance towards inoculating the whole of the poor at their own habitations, for Small-pox or Cow-pox.

In a matter of fuch importance, none, as good men and good fubjects, would refufe; on the contrary, a degree of emulation would be excited, highly beneficial; all would be anxious to participate in fo glorious a work: Thus would the caufe of humanity be ferved, and the beft interefts of the country promoted, by adding to the population of it. An eftimate may be formed of the immenfe number of lives which would be yearly faved, were the *variolous inoculation only employed*; when we recollect that Baron Dimfdale, whofe practice muft have been very extenfive, has informed us, *that he did not lofe a fingle patient in the courfe of twenty years from inoculation.*

At the end of every year, each individual fhould be requefted by the veftries to deliver in an account of the number inoculated, with names and places of abode, within the year, and for which they have received no fort of remuneration: Thefe accounts fhould be publifhed for the information of the parifhioners, and a fubfcription might then be propofed by the veftries, which would enable them to tender to their profeffional brethren, fomething more than the thanks of the parifh for their laudable undertakings.

If fomething like this plan fhould be adopted, inftead of between two and three thoufand who die annually in London of Small-pox, the mortality of this difeafe would be reduced to a cypher. I am, &c.

*Smith Street, Weftminfter,*

*Dec. 10, 1800.*

JOHN FRANKS.

